# Effect of Age at Diagnosis on the Prognosis of Gastric Cancer Patients: A Population-Based Study in Georgia

**DOI:** 10.7759/cureus.62154

**Published:** 2024-06-11

**Authors:** Saba Zhizhilashvili, Irakli Mchedlishvili, Natalia Jankarashvili, Rolando Camacho, Nana Mebonia

**Affiliations:** 1 Epidemiology and Biostatistics, Tbilisi State Medical University, Tbilisi, GEO; 2 Radiation Oncology, Fridon Todua Medical Center, Tbilisi, GEO; 3 Oncology (Non-communicable Diseases), World Health Organization, Mallorca, ESP

**Keywords:** cox proportional hazards regression, predictor factor, survival, gastric cancer, cancer registries

## Abstract

Introduction: The national burden of gastric cancer (GC) is high in Georgia, which is determined by its high mortality and low survival. The study aimed to estimate the effect of age at diagnosis on the prognosis of GC patients diagnosed between 2015 and 2020 in Georgia.

Materials and methods: We obtained data for the study from the national population-based cancer registry. All patients 15 years of age or older, diagnosed during 2015-2020 with invasive GC (site codes C16.0 to C16.9, International Classification of Diseases for Oncology), were eligible for inclusion in the analysis. We produced survival curves using the Kaplan-Meier method, and the log-rank test was used to compare survival between groups. Hazard ratios (HR) were estimated using univariate Cox proportional models and multivariate Cox proportional hazard models. The endpoint of the study was overall survival (OS). The level of statistical significance of the study findings was estimated using p-values and 95% confidence intervals (CI). A p-value<0.05 was considered statistically significant.

Results: A total of 1,828 gastric cancer cases were included in the statistical analysis. The average age of patients was 65 years. The bivariate Cox’s regression analysis demonstrated that the risk of gastric cancer mortality increased gradually with the age of cancer patients. The HR and 95% CI were as follows: 1.5 (1.1-1.8) and 2.1 (1.5-2.5) in the 46-65 years and >65 years groups, respectively, with the <46 years group as a reference. Moreover, multivariable Cox’s regression analysis proved that age is an independent risk factor for GC mortality (HR = 1.4; 95% CI = 1.2-1.8; p<.001).

Conclusion: We found that age at diagnosis was a significant predictor of the worse survival of GC patients diagnosed between 2015 and 2020 in Georgia.

## Introduction

Gastric cancer (GC) is the fifth-most common cancer worldwide. It is a leading cause of cancer deaths and a major public health issue. Global GC incidence and mortality rates have been declining over the last five decades; this may partially be due to the eradication of *Helicobacter pylori* (*H. pylori*), infection, early diagnoses, and advances in treatment [1-6].

Important predictive factors for GC prognosis include tumor patterns at diagnosis and access to effective treatment [2, 7-11]. Significant patient characteristics that can affect GC survival include the patient’s age at diagnosis [7, 8, 12-19] and the presence of comorbidities [8, 20, 21]. Elderly patients with gastric cancer are generally recognized as having a worse long-term prognosis than younger patients [3,4,8,12,14,16], but controversies exist regarding the impact of age on cancer-specific mortality [2].

In 2015-2020, the annual incidence of GC remained stable in Georgia; it was close to 10 per 100,000 people, with the incidence higher among men; recent estimates are 16.0 per 100,000 [22]. Unequal sex distribution, which is a general feature of GC, is clearly demonstrated in Georgia; for example, in 2020, GC morbidity and mortality rates were double in men than in women. Sex differences in GC incidence rates in the country could be explained by lifestyle patterns; it is well established that there is an unequal distribution of non-communicable risk factors between persons of different sexes. Men are more likely to smoke than women (51.5% and 6%, respectively) and are almost six times more likely (29% and 5%, respectively) to report heavy episodic drinking in the last 30 days [23, 24]. Only 8.4% of the Georgian population indicate positive salt intake behaviors (never adding salt to meals and limiting processed foods to reduce salt intake), which is less common among males than females (6.4% and 10.2%). However, fruit and vegetable consumption, an important component of a healthy diet, is equally low in both sexes, with only 37.3% meeting the WHO recommendation of 400 grams of fruit and vegetables per day [25].

The bacterium *H. pylori*, well-known as a leading cause of gastric cancer, is very prevalent in Georgia; more than 70% of adults are infected with *H. pylori* [26]. In addition, previous studies show that about 72% of the patients undergoing endoscopy were histologically positive for *H. pylori* [27].

The national burden of GC is due to its high mortality-more than 500 (13.5/100,000 population) annual deaths, making it the third most common cause after lung and breast cancer. The five-year overall survival (OS) rate of GC was less than 25% during the period 2016-2020 [22].

The study aimed to estimate the effect of age at diagnosis on the prognosis of GC patients who were diagnosed between 2015 and 2020 in Georgia.

## Materials and methods

Study setting and design

We conducted a retrospective cohort study using data from the national population-based cancer registry of Georgia. The study protocol was approved by the Ethics Committee of Tbilisi State Medical University, Tbilisi, Georgia (approval number: 6-2020/83). The written informed consent form was not obtained as the study design did not require personalized information and a non-personalized database was used during statistical analysis. 

Participants

All patients 15 years of age or older, diagnosed during 2015-2020 with invasive GC (site codes C16.0 to C16.9, International Classification of Diseases for Oncology), were eligible for inclusion in the analysis. Cases with in situ stages (1.5%) and incomplete data (2.5%) were excluded from the analysis.

Variables included in the study

We extracted the following variables from the cancer registry database: sex, age at diagnosis, stage at diagnosis, year of diagnosis, and the year of death. The study participants were divided into the following three age groups: a young group (under 46 years), a middle-aged group (46-65 years), and an elderly group (over 65 years). The stage of cancer at diagnosis (I, II, III, IV, and unknown) was collected for each patient using cancer registry data.

Study endpoint

Any cause of death of cancer patients was considered an outcome measure; thus, the OS and not GC-specific survival were counted as the endpoints for the study. Patients’ observation period included the time from the date of diagnosis to the end of the study (August 31, 2022) for those who were alive and the date of death (outcome) for those who died during the follow-up period. The vital status of registered cancer patients is updated in real-time by the linkage between the cancer registry and the national mortality database.

Statistical analysis

For descriptive statistics, categorical variables were presented as numbers and percentages (n, %). Continuous variables were expressed as the mean±standard deviation (SD). To compare categorical variables, the chi-square test, or Fisher’s exact test, was used when applicable.

In analytical statistics, we produced survival curves using the Kaplan-Meier method for each possible predictor for OS. The log-rank test was used to compare survival between groups. Hazard ratios (HR) of variables were estimated using univariate Cox proportional models and multivariate Cox proportional hazard models. To estimate the relationship between possible predictive factors and OS, in addition to the age at diagnosis of GC patients, two potential exposures, namely, the stage of cancer at diagnosis and the sex of patients, were included in the analyses. The reference level for each factor was chosen in advance as follows: younger age, stage I at diagnosis, and male. The patient’s age and tumor stage at diagnosis, which showed a statistically significant correlation with HR in univariate analysis, were included in a multivariate Cox proportional HR model. The level of statistical significance of the study findings was estimated using p-values and 95% confidence intervals (CI). A p-value of <0.05 was considered statistically significant. The obtained data were analyzed using IBM SPSS Statistics for Windows, version 23 (IBM Corp., Armonk, NY).

## Results

Characteristics of GC patients

The risk of developing GC increased dramatically with age in Georgia. Out of 1,828 gastric cancer cases included in the statistical analysis, 111 (6.1%) were detected before the age of 46, 786 (43.0%) between 46 and 65 years, and the remaining 931 (50.9%) were over 65 years of age. The mean age of patients with stomach cancer was 65 (SD: 11.8) years; the age range was 16-85 years. Out of the total study participants, 1,147 (63%) were males and 1,440 (78.8%) patients were diagnosed at the III and IV stages (Table 1). 

**Table 1:** Characteristics of gastric cancer patients enrolled in the analyses

Variables	n	%
Sex		
Male	1147	63
Female	681	37
Age at diagnoses		
Median (age range)	66 years	(16-85)
Under 46 years	111	6.1
46 – 65 years	786	43.0
Over 65 years	931	50.9
Stage at diagnosis		
I	89	4.9
II	221	12.1
III	561	30.7
IV	879	48.1
Unknown	78	4.2

Table 2 summarizes the tumor stage at diagnosis of patients with GC by age group at diagnosis. The proportion of GC cases detected at early (I and II) stages was low for all patients diagnosed in different age groups (under 46 years, 46-65 years, and over 65 years), and the small difference that was revealed was not statistically significant (p = 0.18).

**Table 2:** Tumor stage at diagnosis by age groups for patients with gastric cancer diagnosed during 2015–2020

	Stages					p-value
	I n (%)	II n (%)	III n (%)	IV n (%)	Unknown n (%)	
Age groups						0.18
Under 46 years	7 (6.3)	12 (10.8)	31 (27.9)	58 (52.3)	3 (2.7)	
46 – 65 years	40 (5.1)	91 (11.6)	257 (32.7)	366 (46.6)	32 (4.0)	
Over 65 years	42 (4.5)	118 (12.7)	273 (29.3)	455 (48.9)	43 (4.6)	
Total	89	221	561	879	78	1828

Kaplan-Meier survival analyses

**Figure 1:**
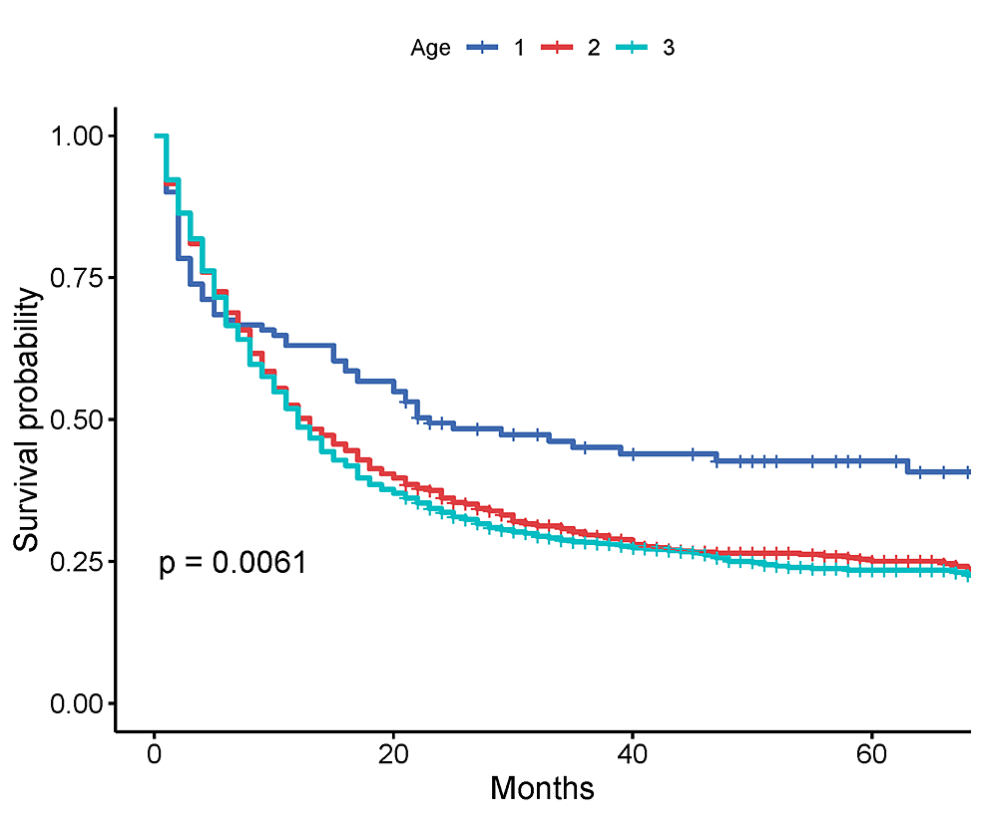
Kaplan-Meier survival by age of patients Age groups: 1: <46 years; 2: 46-65 years; 3: >65 years

On a Kaplan-Meier analysis, the overall median survival for the entire cohort was 13 months (95% CI: 12-15 months). When the cohort was stratified by age groups, the median OS was 23 months (95% CI: 17-60 months), 13 months (95% CI: 11-15 months), and 12 months (95% CI: 11-14 months) for patients under 46 years, 46-65 years, and over 65 years accordingly (p = 0.061, log-rank test) (Figure 1).

**Figure 2:**
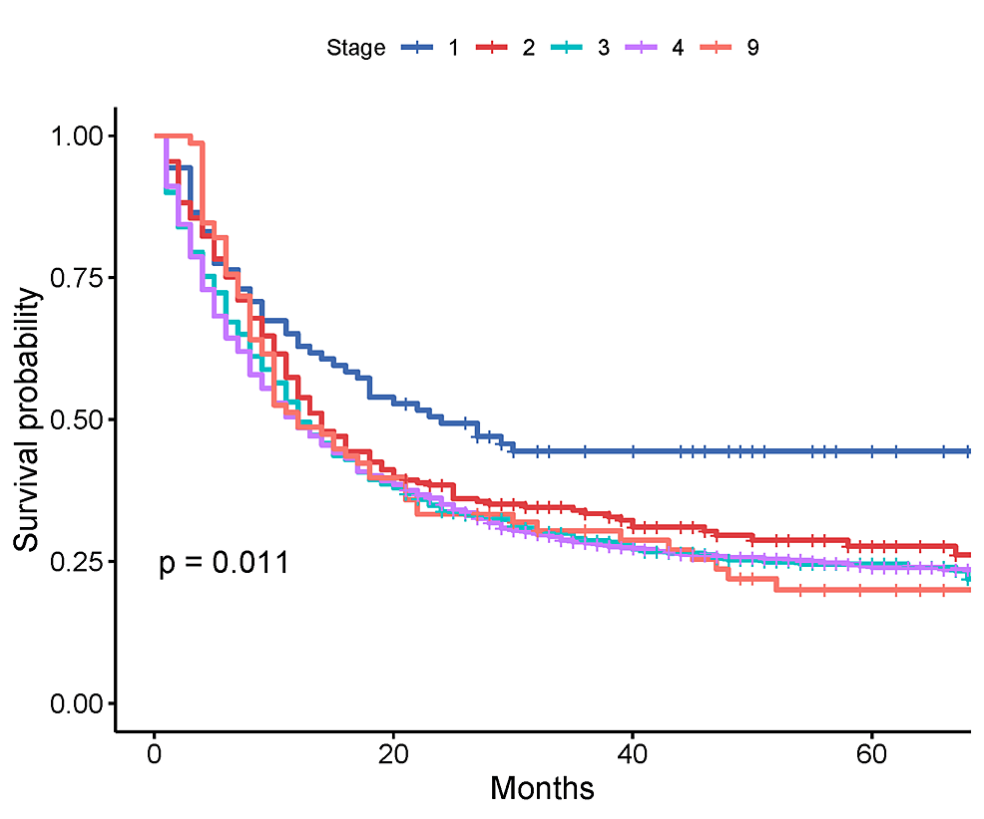
Kaplan-Meier survival by stage of cancer Gastric cancer stages at diagnosis: 1: stage I; 2: stage II; 3: stage III; 4: stage IV; 9: unknown

Stratification by stage groups revealed that the median OS was 24 months (95% CI: 16-60 months), 14 months (95% CI: 12-18 months), 12 months (95% CI: 11-15 months), 12 months (95% CI: 10-14 months), and 12 months (95% CI: 10-21 months) for stage I, II, III, IV, and unknown stage patients, respectively (p = 0.011, log-rank test) (Figure 2).

**Figure 3:**
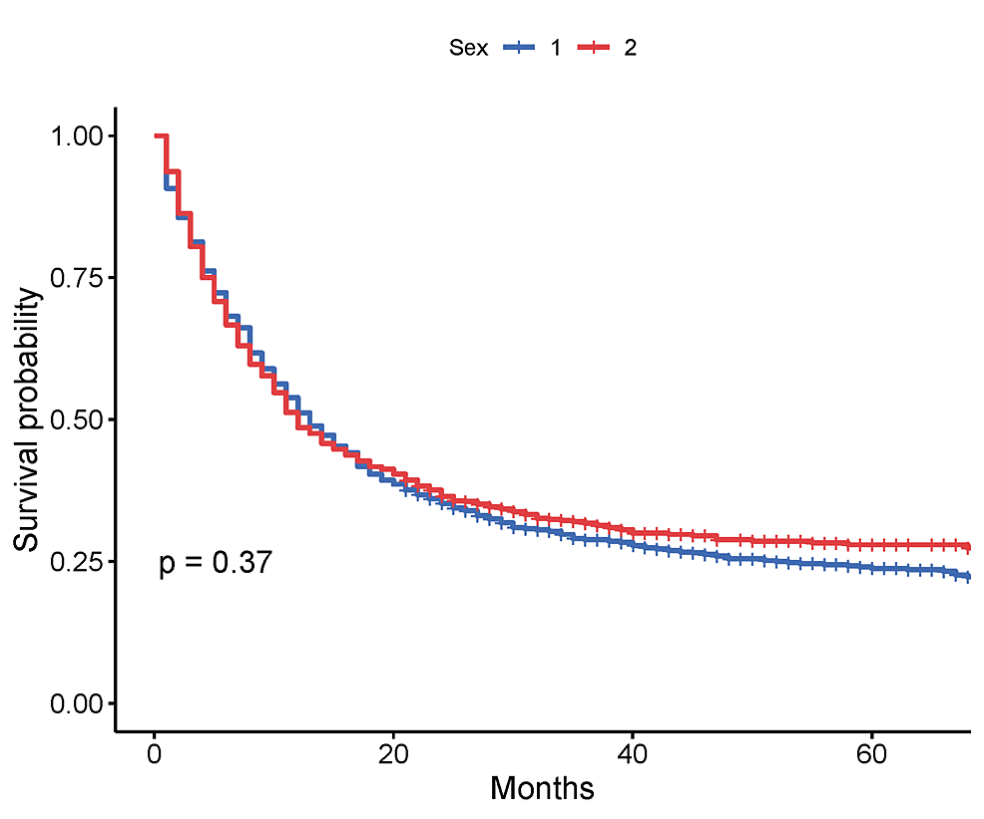
Kaplan-Meier survival by sex of patients Sex of gastric cancer patients: 1: male; 2: female

When male and female patients were compared, the median OS was very similar, with 12 months (95% CI: 11-14 months) and 13 months (95% CI: 12-15 months), respectively (p = 0.37, log-rank test) (Figure 3).

Estimation of the role of potential predictive factors for GCearly mortality

The univariate Cox’s regression analysis demonstrates that the risk of GC mortality increased gradually with the age of cancer patients and was highest over 65 years of age (HR = 2.1; 95% CI: 1.5-2.5). The late stage of cancer at diagnosis was positively and strongly correlated with mortality. The HR for death for cancer detected at the II, III, and IV stages was 1.4 (95% CI: 1.1-1.9), 2.0 (95% CI: 1.5-2.9), and 4.6 (95% CI: 3.4-6.3), times higher than for stage I cancers. The relation of both predictive factors (age and stage) with mortality was statistically significant (p<0.05). The patient’s gender was not significantly correlated with increased mortality (HR = 1.1, 95% CI: 0.9-1.2) (Table 3).

**Table 3:** Hazard ratio (HR) for death from gastric cancer by age at diagnosis, tumor stage, histological grade, and gender (univariate analysis)

Factors	HR	95% CI	p-value
Age at diagnosis			
Under 46 years	-	-	Reference
46 – 65 years	1.5	1.1 - 1.8	0.004
Over 65 years	2.1	1.5 - 2.5	<0.001
Stage at diagnosis			
I	-	-	Reference
II	1.4	1.1 – 1.9	0.050
III	2.0	1.5 – 2.9	<0.001
IV	4.6	3.4 – 6.3	<0.001
Unknown	2.3	1.6 – 3.3	0.020
Sex			
Female	-	-	Reference
Male	1.1	0.9 – 1.2	0.32

In the multivariable Cox’s regression statistical analysis, two variables were enrolled (patients’ age and tumor stage during diagnosis) and they showed a statistically significant association with HR in univariate analyses. The analyses proved that age is an independent statistically significant predictor of high mortality and, consequently, poor survival in patients diagnosed with GC (HR = 1.4, 95% CI: 1.2-1.8) (Table 4).

**Table 4:** Hazard ratio (HR) for death from gastric cancer by stage of tumor and age of patients at diagnosis (multivariable analysis)

Factors	HR	95% CI	p-value
Stage I	-	-	Reference
Stage II	1.4	1.0 – 1.9	.003
Stage III	2.1	1.5 – 2.8	<0.001
Stage IV	4.6	3.4 - 6.3	<0.001
Stage unknown	2.3	1.6 - 3.2	.039
Age	1.4	1.2 - 1.8	<0.001

## Discussion

In this retrospective observational study, we focused on assessing the effect of age at diagnosis on the prognosis of GC patients. The reference age groups of patients used by different researchers vary across the studies, with different cutoff ages to define young, middle-aged, and older patients [8, 21]. Considering the low life expectancy in Georgia, we divided stomach cancer patients into three groups as follows: young (under 46 years), middle-aged (46-65 years), and elderly (over 65 years) groups. The study results were generally consistent with reports in the literature, which demonstrate that younger patients with GC have a better outcome. We detected the highest hazard for mortality in elderly patients. As patients’ age at diagnosis is considered one of the independent factors affecting GC survival [2,7,8,12,14-16], the prognosis of young and elderly patients with stomach cancer has been estimated and compared in various studies, although the results were inconsistent. Most of the investigations found no differences [13,21] or better outcomes [7,8,12,14,16,19], while only a few studies reported poorer survival rates for young patients [17,18]. Also, it has been observed that young patients’ survival was worse due to individual characteristics and different tumor behaviors [8].

Improvements in GC survival have been reported in many counties during the last decades [2,4]. However, there are reports of decreased mortality and improved survival for GC in patients under 75 years of age at diagnosis compared to those over 75 years of age [28]. Differences in stomach cancer mortality and survival and trends between individual countries appear to depend on differences in stage at diagnosis, quality of management, accessibility to healthcare services and treatment approaches, and patients’ characteristics [29-31].

According to our results, the late stage of GC at diagnosis is positively and strongly correlated with poor survival. Cancer stage at diagnosis could be one of the most significant predictors of survival differences between young and older patients. In our data set, the stage of diagnosis varied slightly across different age groups. However, advanced-stage cases predominated in all age groups (Table 2); thus, the disease stage at diagnosis is not able to explain the survival patterns of GC cases detected in different age groups. Previous studies [2, 13] have shown that young patients with GC were most frequently detected at the advanced stage, with metastasis, which is correlated with poor survival, while older patients, diagnosed at early stages, experienced better survival. Furthermore, Lee et al. demonstrated that overall and GC-specific mortalities increased with age in patients diagnosed at I or II stages, although, mortality was not affected by age in patients with late stages of the disease [21].

Early detection of cancer is a big issue in Georgia, even for breast, cervical, and colorectal cancers, for which screening programs have been implemented. The share of all localizations of cancers detected in the early (I and II) stages is slightly more than 50%. The situation is worse in regard to GC where 17.0% of cases enrolled in the analyses were detected at stages I and II, while 78.8% were detected at the III and IV stages, and in 4.2% of cases, the stages were unknown. The reasons for the late detection of cancer and, particularly, GC are multiple. For cancers that are not part of cancer screening programs, special attention should be paid to early diagnosis. Georgia has started early diagnosis programs that focus on raising public awareness and educating primary healthcare physicians about the early symptoms of the most prevalent cancer types. This will facilitate early detection of disease, although intensification of activities in this direction is necessary.

In our study, we found that patient sex was not a statistically significant factor in determining the survival of patients with GC, which is consistent with the literature [11,21].

One additional predictor, which could be the reason for high mortality among elderly GC patients but which we were not able to analyze in our study, is treatment approaches. There is unlimited financial access to GC treatment in Georgia, provided by the Universal Health Coverage (UHC) Program, introduced in 2013 in Georgia, which promotes financial access to health services and has benefited more Georgians, particularly households registered as living under the poverty line and those above the poverty line but earning less than the highest income bracket [32]. Moreover, human resources needed for GC management are readily available in the country. However, the cause of the particularly low coverage of advanced treatments among older patients revealed by the Georgian Cancer Registry could be their health status. Older patients, because of their worse health conditions with comorbidities and poorer tolerance to treatment, mainly receive conservative treatment and are less likely to have advanced chemotherapy and even standardized surgery in comparison to young patients [8,21]. 

A strength of our study is its demonstration of the effective use of a population-based cancer registry. The Georgia Cancer Registry, established in 2015, facilitates the collection of comprehensive data on cancer incidence and mortality, enabling survival analyses and other studies to inform cancer management and prevention policies. In Georgia, cancer registry data quality is monitored with the International Agency for Research on Cancer (IARC) Check Tool. In addition, access to independent sources, such as hospital discharge registries, social care agency databases, and national mortality databases, allows cross-checking the quality of cancer registry data.

Among the limitations, we acknowledge that we did not analyze the role of treatment approaches in the prognoses of study participants. Treatment methodologies used in different age groups might have influenced the outcome differences revealed among studies [13]. Another limitation is that we did not estimate the role of comorbidities in the survival of GC patients. These limitations should be considered in future investigations.

## Conclusions

Our population-based study indicates that older age at diagnosis is a statistically significant predictor for the worse survival of GC patients, diagnosed during 2015-2020 in Georgia. We found that the proportion of stages of GC diagnosed in different ages was almost the same; thus, the late stage of diagnoses, which prevails in each age group is unable to explain the different survival of patients detected in different age groups. Further studies are needed to identify the reasons for low survival in elderly patients. In addition, approaches to the treatment of older patients with GC in Georgia need to be reviewed and standardized. The reasons for the late detection of cancer and, particularly, GC are multiple. For cancers that are not part of cancer screening programs, special attention should be paid to early diagnosis. Georgia has started early diagnosis programs that focus on raising public awareness and educating primary healthcare physicians about the early symptoms of the most prevalent cancer types. This will facilitate early detection of disease, although intensification of activities in this direction is necessary.
